# A hybrid approach based on the analytic hierarchy process and 2-tuple hybrid ordered weighted averaging for location selection of distribution centers

**DOI:** 10.1371/journal.pone.0206966

**Published:** 2018-11-08

**Authors:** Shilong Li, Zhenlin Wei

**Affiliations:** School of Traffic and Transportation, Beijing Jiaotong University, Beijing, China; Southwest University, CHINA

## Abstract

Distribution centers (DCs) are an important part of the modern logistics system. The selection of a location for a DC is significant for saving costs and reducing externalities caused by distribution. In this paper, we propose a new hybrid method based on the analytic hierarchy process (AHP) and 2-tuple hybrid ordered weighted averaging (THOWA) to select the location of a DC in a megacity. First, we propose a new set of evaluation criteria integrating economic, political, social and ecological information based on the characteristics of Chinese megacities. Second, subjective criteria weights are calculated by AHP combining the evaluation of logistics experts. Third, experts from academia, enterprise and government assess the performance of alternatives. In addition, the overall evaluation values are aggregated by an improved THOWA operator to rank the alternatives. Finally, we conduct a sensitivity analysis to investigate the influence of criteria weights on the decision-making process. The proposed method is novel and addresses the uncertainty under limited quantitative information, which has the advantages of avoiding information loss and distortion problems in the integrating process and operating linguistic evaluation information effectively. The proposed method can be practically applied by municipal planning departments in deciding on the location of new DCs. A numerical application of the proposed method is provided.

## Introduction

With the development of economies and the growth of populations, distribution requirements have grown rapidly, which has inevitably brought opportunities and challenges for urban logistics systems [1]. The distribution center (DC) is an important part of the logistics system, which plays an important role in distributing goods for customers [2]. Location planning for a DC is especially vital for saving costs, improving the efficiency of transport flows, and increasing customer satisfaction [3]. However, municipal planning departments are facing new issues for location planning. For example, the sprawl of DCs has caused increasing exhaust pollution and congestion due to the high price of land and urban planning in megacities [4]. Municipal planning departments should assess the merits and limitations of land use allocation related to DCs [5]. Hence, a study on the location selection of DCs considering the characteristics of megacities has theoretical significance and application value [6].

There are many megacities in China, such as Beijing, Shanghai, Shenzhen, and Guangzhou. The characteristics of megacities have significantly influenced the location selection of DCs, including large numbers of people, serious air pollution [7], traffic congestion [8], sensitive areas to be protected [9], high land prices, high labor prices [10], rigorous urban planning [11], urban customer zones, and developed transportation infrastructures. With the mentioned characteristics, it is clear that the location selection of DCs in the megacities is a complex decision that involves consideration of multiple criteria such as minimum ecological cost, least impact on environment and society, and conformance to the megacity policies. In particular, the characteristics are difficult to obtain. Therefore, the process of location selection is under an uncertainty environment, which increases the complexity of location selection. Moreover, with increasing emphasis on environmental and social responsibility, we must focus on the sustainability of the megacity when selecting the location of DCs.

In this paper, we studied the problem of location selection of DCs from the perspective of the megacities’ sustainability, proposing a hybrid method based on the analytic hierarchy process and 2-tuple hybrid ordered weighted averaging for selecting the alternatives of for DC location. A new set of evaluation criteria integrating economy, policy, environment and ecology based on the characteristics of Chinese megacities is proposed to evaluate the alternatives. AHP is used to calculate criteria weights combining the evaluation of logistics experts. Fuzzy set theory is utilized to assess the performance of alternatives due to the lack of numeric information. THOWA is employed to aggregate the overall evaluation values to rank those alternatives. Our contribution is a hybrid method based on AHP and THOWA for the location selection of DCs to help municipal planning departments select the optimal location for a DC considering sustainability.

The remainder of this paper is organized as follows. The ‘Literature review’ section reviews the literature on the location selection of DCs. The ‘Selection of location criteria’ section proposes the criteria and subcriteria for evaluating alternatives from the perspective of the economy, policy, society and ecology. The ‘Methods’ section presents an introduction of the method based on AHP and the improved THOWA. The ‘Numerical illustration’ section presents a numerical application to verify the availability of the method. The ‘Sensitivity analysis’ section conducts a sensitivity analysis to investigate the influence of criteria weights on the decision-making process. The final section concludes the paper.

## Literature review

The problem of location selection of urban DCs can be classified as a special case of the facility location problem [3, 6]. In recent years, several researchers have conducted studies on location planning and evaluation of facilities. The most common approaches can be divided into quantitative methods and the qualitative methods [12]. Quantitative calculations are applied to a certain and deterministic environment with certain numerical dates for the description of the alternatives. In addition, intelligent heuristic algorithms are usually applied to solve the established model. For example, Ahmed et al. [13] developed an efficient genetic algorithm which was capable of solving a very large-scale mixed integer linear programming problem of a reverse logistics network. Abdelhalim et al. [14] addressed a location-inventory-routing model for perishable products and applied a genetic algorithm approach to solve the problem efficiently. Rahmani et al. [15] proposed a new hybrid optimization method called the hybrid evolutionary firefly genetic algorithm to solve the capacitated facility location problem. Eliana et al. [16] developed a new mathematical model considering the minimization of operational costs and the minimization of environmental effects to solve the capacitated location-routing problem. Mohammad et al. [17] presented a new multiobjective model for a hub location problem under uncertainty and proposed a hybrid two-phase solution method to solve this model. John et al. [18] proposed a two-phase hybrid heuristic algorithm to solve the capacitated location-routing problem. Xiang et al. [12] built a model with constraints that satisfies the demand of each customer and minimizes the distance cost and proposed a novel approach of the adaptive particle swarm optimization (APSO) algorithm for solving this model. Kannan et al. [19] designed a multiechelon, multiperiod, multiobjective model for a sustainable reverse logistics network, for which a customized multiobjective particle swarm optimization algorithm was applied to obtain solutions on the Pareto frontier.

Notably, in the actual location selection process, many numerical datasets of the alternatives are difficult to obtain with certainty due to the complexity and ambiguity of the decision environment. For such situations, the usage of quantitative methods to solve the location selection problem is limited. To address the decision of the location of DCs under an uncertain environment, the qualitative method has been proposed and applied [20]. For example, Yan et al. [1] presented a new hybrid fuzzy multiple-criteria decision-making method for selecting the location of a joint distribution center under limited quantitative information for the alternatives. Anjali et al. [3] used fuzzy theory to quantify criteria values under uncertainty and applied fuzzy TOPSIS to evaluate and select the best location for implementing an urban distribution center. Balaram et al. [21] utilized the group heterogeneity concept based on a pairwise comparison approach of the decision parameters to extract the realistic and relatively more accurate information assessed by the researchers on this territory. Tufan et al. [22] applied a multicriteria Choquet integral method to capture the imprecise or vague nature of qualitative criteria, which showed a successful application in a real warehouse location selection problem. Rao et al. [6] presented a fuzzy multiattribute group decision making technique based on a linguistic two-tuple method to evaluate potential alternative city logistics center locations from a sustainability perspective. Mahamaya et al. [23] developed a systematic and integrated fuzzy decision analysis-oriented model based on fuzzy-TISM (total interpretative structural modeling), which focuses on enhancing sustainability by addressing issues of the strategic decision in an uncertain environment. Finally, a summary of available methods for location selection of DCs is given in Table 1, which classifies the category of the method, the goal and algorithm of the model of the quantitative method and the evaluation criteria and information aggregation algorithm of the qualitative method.

**Table 1:** Summary of available methods for the location selection of DCs.

Category	Author and Literature	Goal or Criteria	Algorithm
**Quantitative method**	Ahmed Alshamsi [13]	Maximize efficiency	Genetic algorithm
	Abdelhalim Hiassat [14]	Minimum total cost	Genetic algorithm
	A. Rahmani [15]	Minimum total cost	Hybrid evolutionary Firefly genetic algorithm
	Eliana M. Toro [16]	Minimum operational costs and minimum environmental effects	Classical epsilon constraint technique
	Mohammad Zhalechian [17]	Maximize responsiveness	Self-adaptive differential evolution algorithm
	John Willmer Escobar [18]	Minimum total cost	A two-phase hybrid heuristic algorithm
	Xiang Hua [12]	Minimum value of the sum of the demand and distance	Adaptive particle swarm optimization algorithm
	Design Kannan Govindan [19]	Minimum cost and environment effect, maximize social responsibility	Multiobjective particle swarm optimization algorithm
	Stefan Treitl [28]	Trade-off between total cost and carbon emission	CPLEX
**Qualitative method**	Yandong He [1]	Economic, environmental, and social	Hybrid fuzzy multiple-criteria decision-making method
	Balaram Dey [21]	Economic, and social	A new Multicriteria group decision-making approach
	Anjali Awasthi [3]	Economic, environmental, and social	Fuzzy TOPSIS
	Tufan Demirel [22]	Economic, and social	Choquet integral
	Congjun Rao [6]	Economic, environmental, and social	2-Tuple hybrid ordered weighted averaging operator
	Mahamaya Mohanty [23]	Economic, environmental, and social	Fuzzy-TISM (total interpretative structural modeling)
	Thi Yen PHAM [24]	Economic, environmental, and social	Fuzzy-Delphi approach
	Sana Malik [25]	Economic, environmental, and social	Graph theory and matrix approach
	Jacek ZAK [26]	Economic, environmental, social, and technological	Multiple criteria decision making/aiding methodology
	Sen Guo [27]	Economic, environmental, and social	Fuzzy TOPSIS

The traditional criteria employed in the quantitative methods have predominantly focused on cost and surrounding conditions. However, with the emphasis on environmental protection, many researchers have gradually begun to consider environmental criteria in their studies. For instance, Thi et al. [24] studied the problem of logistics center location by considering the environmental impact of construction activities and transportation activities, which simultaneously has negative impacts on the city residents. Sana et al. [25] selected the most appropriate location for a collection center from the perspective of sustainability, considering both environmental and social criteria in the evaluation system. Jacek et al. [26] performed multiple criteria evaluation system in the selection of the most desirable location of the logistics centers, which contain economic, infrastructural, technological, social and environmental potential. Sen et al. [27] studied selecting the most sustainable site for electric vehicle charging stations and built an evaluation index system from a sustainability perspective, which consists of economic, social, and environmental criteria associated with a total of 11 subcriteria. Stefan et al. [28] simultaneously considered environmental sustainability factors and total cost when selecting the location for a facility, in which transport carbon emissions was identified as the main environmental impact factor.

However, the political factor, which has an important influence on the location selection of DCs in Chinese megacities, has been ignored by the vast majority of researchers. In China, the government determines the direction of the economy through urban planning and incentives policies. Economic activities that oppose the political direction will inevitably be prohibited. Hence, we must address the political criteria when designing an evaluation criteria system for the location selection of DCs.

In this paper, we designed an evaluation criteria system based on the characteristics of Chinese megacities by the integration of economic, political, social and ecological dimensions. Furthermore, we propose a new hybrid method based on AHP and THOWA for location selection of DCs. The proposed approach is novel and can be applied with limited quantitative information, and provides a practical method for location selection of DCs for municipal planning departments in Chinese megacities.

### Selection of location criteria

As many factors have an impact on location selection, the location selection of DCs can be seen as a multicriteria decision-making (MCDM) problem [3, 6, 29]. In this paper, the criteria are acquired from literature reviews, analysis of megacities’ characteristics, and opinions of logistics experts who have worked in the enterprise, government, or academia for at least ten years. The final list contains 4 criteria and 12 subcriteria (as shown in Table 2), which will be used to evaluate and select the optimal location of a DC from a sustainability perspective.

**Table 2:** List of evaluation criteria system.

Criteria category	Id	Subcriteria	Type	Sources
Economic	C1	The price of land	Cost	[1, 3, 6, 21, 25]
	C2	Labor criteria	Benefit	[3, 6, 22, 25]
	C3	Customer distribution	Benefit	[3, 6, 21, 24]
Political	C4	City planning	Benefit	Our team
	C5	Incentive policy	Benefit	[22, 24]
Social	C6	Traffic conditions	Benefit	[3, 6, 21, 22, 24, 26]
	C7	Public facilities conditions	Benefit	[6, 22]
	C8	Impact on the surroundings	Cost	[1, 6, 23, 27]
	C9	Impact on traffic congestion	Cost	[1, 6]
Ecological	C10	Natural conditions	Benefit	[6]
	C11	Pollutant emission	Cost	[6, 23, 24, 27]
	C12	Sensitivity to pollution	Benefit	Our team

### Economic criteria

The price of land (C_1_): Land is an important foundation for the construction of a DC, and is required for the operation of a DC [6]. However, the prices of land are very high in megacities, especially in urban areas. The more money enterprises spend on land, the heavier the financial burdens they will have [1, 3].

Labor criteria (C_2_): As many technologies have been applied to the operation of a DC, many professional operators are required. In addition, operations such as handling, packaging and storage still require many laborers [3], which makes it necessary to have a sufficient number of laborers in the vicinity of the alternative. Moreover, the level of wages must be considered.

Customer distribution (C_3_): The purpose of building a DC is to serve customers conveniently. Thus, the distance between the customers and the DC should be as short as possible to ensure that customers can receive their delivery service in time [1]. As a result, the distribution costs will be reduced in this case.

### Political criteria

City planning (C_4_): "City planning" is the comprehensive deployment of a reasonable layout and the overall arrangement of the city’s construction projects for the future development of a city. DCs are an important component of the urban infrastructure. Therefore, the construction of DCs should conform to city planning.

Incentive policy (C_5_): When selecting the location of a DC, we should conduct a comprehensive investigation of the local incentive policy, such as the land policy, tax policy and financial policy [22]. With favorable investment policies and environment, distribution enterprises can acquire land more easily, reduce tax costs and obtain better financial support [24].

### Social criteria

Traffic conditions (C_6_): Convenient traffic conditions are beneficial for facilitating distribution activities, saving on operating costs, improving the service level, and enhancing the competition ability [24]. Therefore, the DC should connect with multiple modes of transportation, e.g., highway, urban roads, airports, seaports and railways, to facilitate transit [3].

Public facilities conditions (C_7_): Public facilities are significant factors that support the operation of a DC. The vital related public facilities are as follows: networks, communication, electricity and water supply [6, 22].

Impact on the surroundings (C_8_): The operation of the DC, such as loading and unloading, handling and packing, will produce noise that has a negative impact on nearby areas [23]. Moreover, as the DC is the key fire protection unit, the surrounding buildings can easily ignite once the DC catches fire. Therefore, the location should maintain an appropriate distance from sensitive areas, such as cultural heritage sites and residential quarters.

Impact on traffic congestion (C_9_): Traffic congestion has become a dilemma for megacities [6]. As is known, the longer the delivery distance is, the more the delivery vehicles will contribute to congestion. Therefore, a suitable location minimizes the delivery flow as much as possible to reduce the impact on traffic congestion [1].

### Ecological criteria

Natural conditions (C_10_): The location selection of the DC must consider the local natural conditions [6], e.g., meteorological conditions, geological conditions, hydrological conditions, and topographic conditions. All of those have impacts on the normal operation of a DC.

Pollutant emissions (C_11_): Most of the delivery vehicles are internal-combustion engine vehicles whose exhaust emissions have a negative impact on the environment and human beings [24, 27]. The amount of pollutants is directly related to the distribution distance. Hence, when selecting the location of a DC, the level of distribution distance must be considered.

Sensitivity to pollution (C_12_): The areas that are sensitive to the environment require higher environmental quality, such as scenic spots, cultural heritage sites, and drinking water source protection areas. Due to the pollution caused by the operation of a DC, the location should not be in proximity to those sensitive areas.

Further, the above 12 subcriteria can be classified as either a cost type or a benefit type based on their economic characteristics. Subcriteria C_1_, C_7_, C_8_, C_11_ are cost type attributes, whose evaluation values are “Very low, Low, Lower-middle, Middle, Upper-middle, High, and Very high”. Particularly, the smaller the evaluation value, the better the corresponding DC location. Subcriteria C_2_, C_3_, C_4_, C_5_, C_6_, C_9_, and C_10_ are benefit attributes, whose evaluation values are “Very poor, Poor, Lower-middle, Middle, Upper-middle, Good, and Very good”. In addition, the higher the evaluation value, the better the corresponding DC location.

## Methods

**Fig 1:**
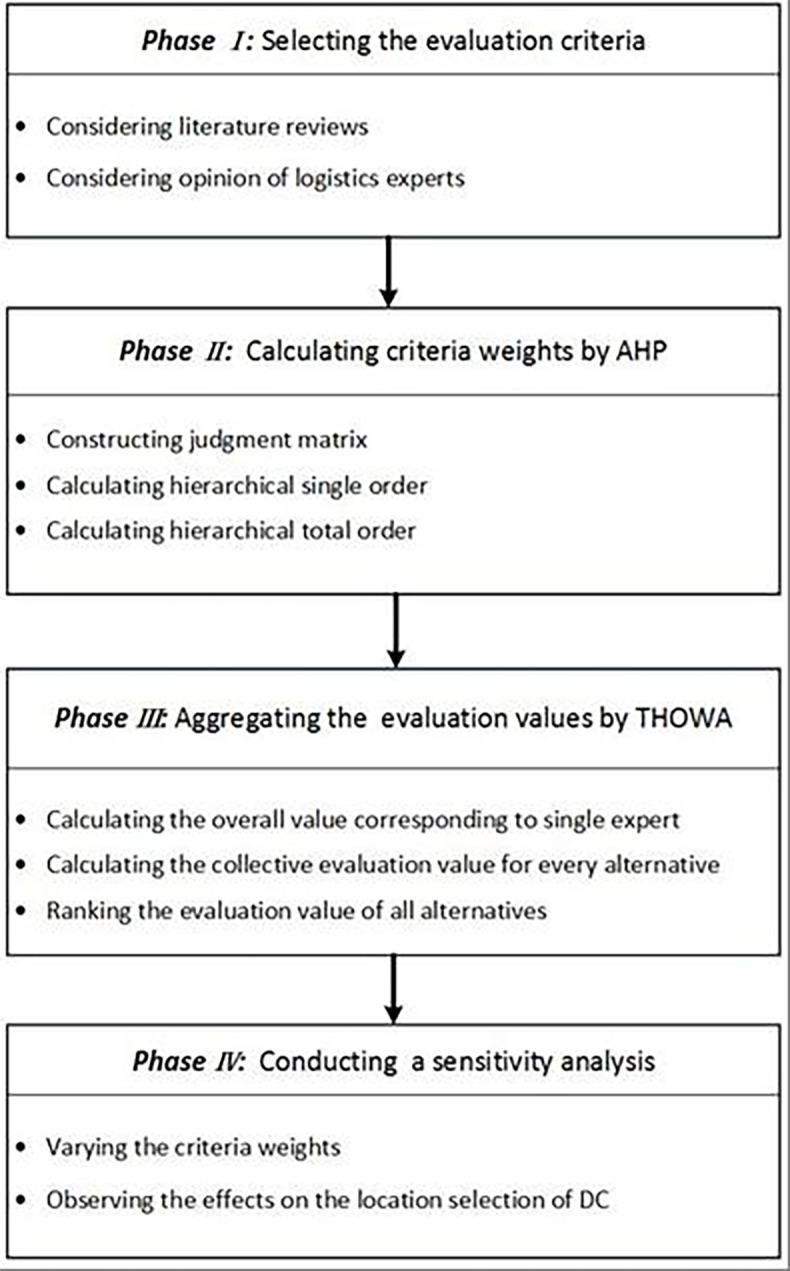
The schematic diagram of the research methodology.

In this paper, a four-phase research methodology was proposed to evaluate and select optimal alternative locations for a DC. In phase 1, the evaluation criteria system was established by considering literature reviews and the opinion of logistics experts. In phase 2, AHP was used to calculate the criteria weights combining the evaluation of logistics experts. In phase 3, THOWA was used to aggregate the overall evaluation values to rank those alternatives. In phase 4, a sensitivity analysis was conducted to investigate the effect of the criteria weights on the location selection of the DC. To better describe our methodology, a framework was constructed [30, 31], as shown in Fig 1.

### Problem hypothesis

Suppose a megacity plans to establish a new DC, the location of which needs to be selected from m alternatives. The set of potential DC locations is denoted as *A* = {*A*_1_,*A*_2_,⋯*A*_*m*_}. The 12 evaluation criteria used to evaluate the alternatives are denoted as *C* = {*C*_1_,C_2_,⋯,C_12_}, whose corresponding weights are denoted as w = {*w*_1_,*w*_2_,⋯,*w*_12_}, with 0≤*w*_*j*_≤1 and $\sum_{j=1}^{12}w_{j}=1$.

Suppose *l* experts participate in the evaluation process, who are denoted as *E*_*k*_(*k* = 1,2,⋯,*l*). Their weight vectors are *v* = {*v*_1_,*v*_2_,⋯,v_k_}, *k* = 1,2,…,*l*, with 0≤*v*_*k*_≤1 and $\sum_{k=1}^{l}v_{k}=1$. The evaluation for an alternative *A*_*i*_(*i* = 1,2,⋯,*m*) with respect to criteria C_j_(j = 1,2,⋯,12) given by expert *E*_*k*_(*k* = 1,2,⋯,*l*) is denoted as a fuzzy language variable $r_{ij}^{k}$. In addition, the corresponding original decision matrix is $R_{k}={\left(r_{ij}^{k}\right)}_{i\times 12},(k=1,2,\cdots ,l)$.

### AHP

To calculate the weights of the subcriteria, the analytic hierarchy process (AHP) is employed. AHP was proposed in the early 1970s by Saaty [32] for evaluating and selecting the optimal scheme against the criteria. AHP can solve complex problems in systems engineering that are difficult to effectively analyze by quantitative methods. In addition, it is a method widely applied in different engineering tasks, such as risk assessment [33], fuzzy fault tree analysis [34], rating the condition and performance of transit infrastructures [35], and selecting location of logistics centers [29, 36, 37]. The application steps of AHP are described simply as follows:

**Step 1:** Establish the structure of the evaluation system. The structure contains three hierarchies: the top level (objective), the interlayer (criteria), and bottom level (subcriteria).
**Step 2:** Construct the judgment matrix. To calculate the weight of each element relative to the previous hierarchy, the 1~9 scale method is introduced [32]. The factor *a*_*ij*_ in the matrix indicates the relative importance of the *i*_*th*_ factor compared to the *j*_*th*_ factor. Then, the factors in the same hierarchy are compared to obtain their judgment matrix.
**Step 3:** Calculate hierarchical single order. To obtain the weight of each element for the previous hierarchy, calculate the maximum eigenvalue (*λ*_max_) and eigenvector of the judgment matrix by the arithmetic average method with the MATLAB software, and normalize the factors in the eigenvector.
**Step 4:** Check the consistency of the hierarchical single order. Calculate the consistency index *CI* where $CI=\frac{\lambda_{\max}-n}{n-1}$, and n is the matrix size. Divide CI by RI (the average random consistency index of the judgment matrix [32]) to obtain the CR. If CR=CI/RI<0.1, then the consistency of the judgment matrix is satisfied. Otherwise, the judgment matrix should be adjusted or eliminated until satisfactory consistency is achieved.
**Step 5:** Calculate the hierarchical total order. To calculate the weights of the subcriteria (w = {*w*_1_,*w*_2_,⋯,*w*_12_}) for the objective, the same process described above is used to calculate the weights of the criteria of the lower hierarchy for the previous hierarchy step-by-step. Then, the hierarchical total orders are obtained.
**Step 6:** Check the consistency of the hierarchical total order. Let CI_j_ be the single ranking consistency index of the hierarchical factors relative to the previous hierarchy, and *RI*_*j*_ is the corresponding average random consistency index. Then, the consistency ratio of the hierarchy total order is calculated as follows:

$$CR=\frac{\sum_{j=1}^{m}p_{j}CI_{j}}{\sum_{j=1}^{m}p_{j}RI_{j}}$$ (1)

If CR<0.1, then the calculation of the previous step is satisfactory.

### THOWA

Since the evaluation results given by experts are fuzzy linguistic variables, we should employ a method to transform those into the form of fuzzy numbers to handle the fuzzy linguistic variables [38, 39]. THOWA is a kind of information processing method proposed by Herrera [40], and characterized by the use of 2-tuple linguistic variables to express and compute the linguistic evaluation information. The strength of the model is the ability to avoid information loss and distortion problems in the process of integrating and operating linguistic evaluation information effectively, thus ensuring that the calculation results are more accurate [40]. The justification, definitions, and theorems of the 2-tuple hybrid ordered weighted averaging model can be seen in these studies [6, 41, 42]. In addition, the steps for the method are as follows:

**Step 1:** Let *S* be a predefined ordered natural linguistic variables evaluation set consisting of odd linguistic fuzzy variables *S* = (*s*_0_,*s*_1_,⋯,*s*_*t*−1_). In this paper, the value of t is 7, and *S* = (*s*_0_,*s*_1_,⋯,*s*_6_) is defined as follows:
  1. For the benefit criteria, define S={s_0_=Very poor, s_1_=Poor, s_2_=Lower-middle, s_3_=Middle, s_4_=Upper-middle, s_5_=Good, s_6_=Very good}.
  2. For the cost criteria, define S={s_6_=Very low, s_5_=Low, s_4_=lower-middle, s_3_=Middle, s_2_=Upper-middle, s_1_=High, s_0_=Very high}.

Construct the original decision matrix *R*_*k*_ with the evaluation linguistic fuzzy variables given by experts, then convert any element in the decision matrix into a linguistic 2-tuple to construct the linguistic decision matrix, such as converting $r_{ij}^{k}$ into $\left(r_{ij}^{k},0\right)$. Thus, the linguistic 2-tuple decision matrix *R*^*k*^ is constructed.

**Step 2:** Aggregate all evaluation values of alternative *A*_*i*_ in *R*^*k*^ by the TWA operator [6] to obtain the evaluation value $y_{i}^{k}$, which represents the overall evaluation of the alternative *A*_*i*_ corresponding to the expert *E*_*k*_, that is,

$$y_{i}^{k}=(s_{i}^{k},\alpha_{i}^{k})=TWA_{w}((r_{i1}^{k},0),(r_{i2}^{k},0),\cdots ,(r_{i12}^{k},0))=\Delta (\sum_{j=1}^{12}w_{j}(r_{ij}^{k},0)),k=1,2,\ldots ,l$$ (2)

**Step 3:** Aggregate the overall evaluation value $y_{i}^{k}$ (*k* = 1,2,…,*l*) corresponding to expert *E*^*k*^ by the THOWA operator [6] to obtain the overall evaluation of the alternative *A*_*i*_,*i* = 1,2,⋯,*m*.

$$y_{i}=(s_{i},\alpha_{i})=THOWA_{\omega }((s_{i}^{1},\alpha_{i}^{1}),(s_{i}^{2},\alpha_{i}^{2}),\cdots (s_{i}^{l},\alpha_{i}^{l}))=\Delta (\sum_{k=1}^{l}\omega_{k}\Delta^{-1}({\dot{s}}_{i}^{\tau (k)},{\dot{\alpha }}_{i}^{\tau (k)}))$$ (3)

where *ω* = (*ω*_1_,*ω*_2_,⋯,*ω*_*l*_) is a position weighted vector, with 0≤*ω*_*k*_≤1, and $\sum_{k=1}^{l}\omega_{k}=1$. $\left({\dot{s}}_{i}^{\tau (k)},{\dot{\alpha }}_{i}^{\tau (k)}\right)$ is the *k*_*th*_ largest 2-tuple of the weighted linguistic 2-tuple $({\dot{s}}_{i}^{k},{\dot{\alpha }}_{i}^{k}),k=1,2,\cdots ,l$, and $({\dot{s}}_{i}^{k},{\dot{\alpha }}_{i}^{k})=\Delta (Q\upsilon_{k}\Delta^{-1}(s_{i}^{k},\alpha_{i}^{k})),k=1,2,\cdots ,l$, where *υ*_*k*_ is the weight vector of expert *E*_*k*_, with 0≤*υ*_*k*_≤1, and $\sum_{k=1}^{l}\upsilon_{k}=1$. Q is the balancing coefficient, with $Q={\left(\sum_{k=1}^{l}\omega_{k}\upsilon_{k}\right)}^{-1}$.

The position weighted vector *ω* = (*ω*_1_,*ω*_2_,⋯,*ω*_*n*_) is determined by a weighted decision method based on normal distribution. In this paper, we improved *THOWA*_*ω*_(*S*) by upgrading the calculation method for *ω* [43], which can relieve the influence of unfair arguments on the aggregated results more effectively [43] than the calculation method used in previous studies, such as [6, 44]. In this paper, the weighted vector *ω* is obtained by

$$\omega_{i}=\frac{e^{-\left[{\left(i-\mu_{n}\right)}^{2}/2\sigma_{n}^{2}\right]}}{\sum_{j=1}^{n}e^{-\left[{\left(j-\mu_{n}\right)}^{2}/2\sigma_{n}^{2}\right]}},\;i=1,2,\ldots ,n$$ (4)

where $\mu_{n}=\frac{1+n}{2}$ and $\sigma_{n}=\sqrt{\frac{1}{n}\sum_{j=1}^{n}{\left(j-\mu_{n}\right)}^{2}}$.

*μ*_*n*_ and *σ*_*n*_ are the mean and standard deviation, respectively, of array (1,2,⋯*n*). Particularly, the application of *ω*_*i*_ can reduce the influence of unfair arguments on the aggregated result by assigning lower weights to those “biased” and “false” arguments, thus making the aggregated result more accurate [42].

**Step 4:** Rank the overall evaluation value *y*_*i*_(*i* = 1,2,⋯,*m*) by the linguistic 2-tuple comparison rules [41]. The larger the value of *y*_*i*_, the more suitable the alternative *A*_*i*_. In addition, the alternative with the largest value is the optimal location.

## Numerical illustration

### Problem description

To demonstrate the feasibility and practicability of the proposed method (AHP+THOWA), we give an example of location selection of a DC under a fuzzy information environment.

**Fig 2:**
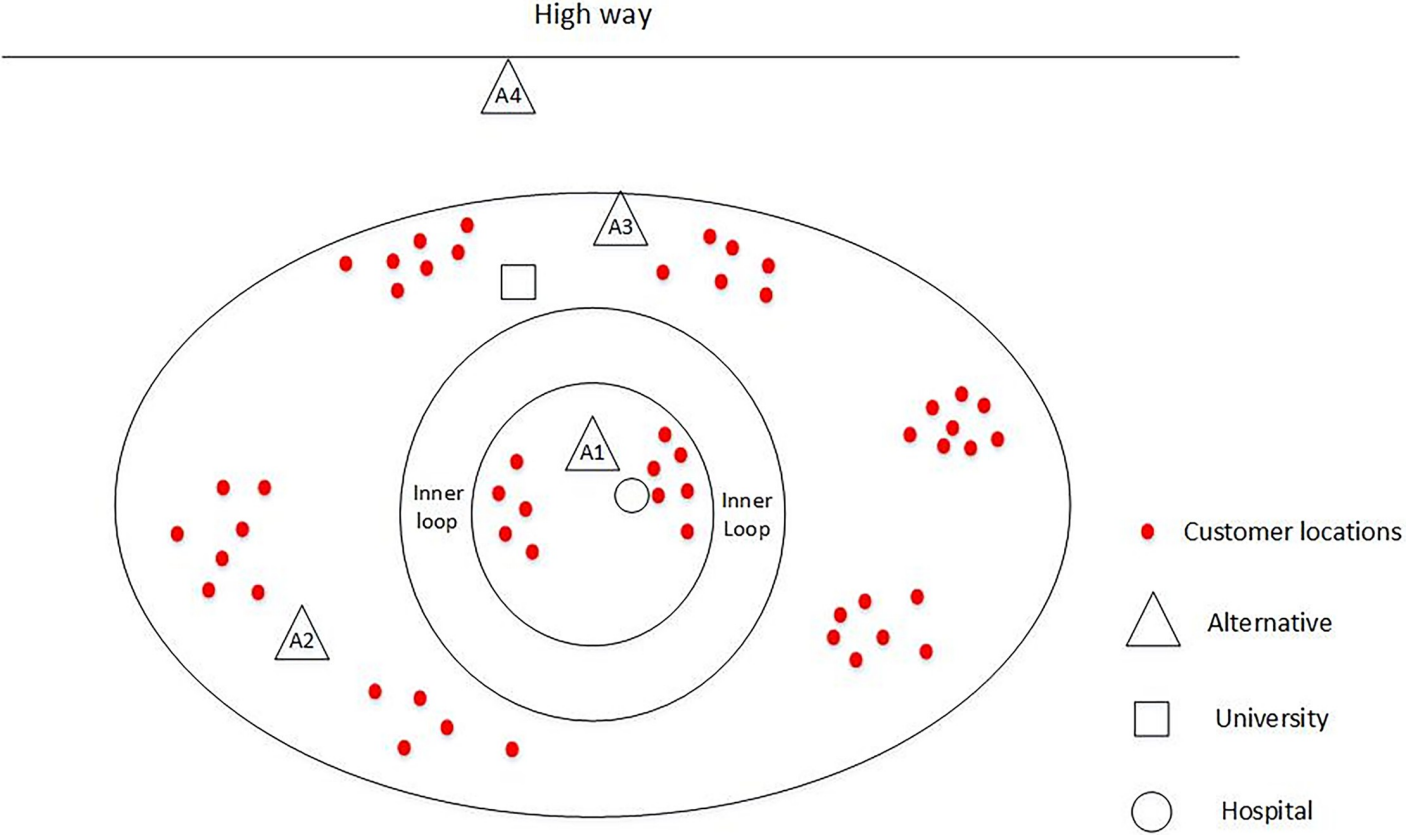
Potential locations for the urban DCs.

Let us assume that the municipal planning department of a megacity plans to build a new DC. There are four alternatives available which are denoted as A_1_, A_2_, A_3_ and A_4_. As shown in Fig 2, we can see that location A_1_ is situated in the city center closest to the customer locations and a hospital (sensitive area) but far from highways; location A_2_ is situated on the outskirts inside the city and far from the highway and sensitive areas; location A_3_ is situated on the outskirts inside the city closest to the highways and a university (sensitive area), and location A_4_ is situated outside the city closest to a highway and far from the customer locations.

In the following section, we use AHP+THOWA to select the optimal alternative for building the DC from A_1_, A_2,_ A_3_ and A_4_.

### Criteria weight generation using AHP

An expert that has a doctorate degree in logistics systems engineering and has been working as an associate professor in academia for over 15 years was invited to make pairwise comparisons of the criteria and subcriteria to determine their relative importance. The results are listed in Tables 3–7.

**Table 3:** Comparison matrix of criteria.

Criteria	Economic criteria	Political criteria	Social criteria	Ecological criteria
**Economic criteria**	1	7	3	5
**Political criteria**	1/7	1	1/5	1/3
**Social criteria**	1/3	5	1	3
**Ecological criteria**	1/5	3	1/3	1

**Table 4:** Comparison matrix of economic criteria.

Economic criteria	C_1_	C_2_	C_3_
**The price of land (C_1_)**	1	5	3
**Labor criteria (C_2_)**	1/5	1	1/3
**Customer distribution (C_3_)**	1/3	3	1

**Table 5:** Comparison matrix of political criteria.

Political criteria	C_4_	C_5_
**City planning (C_4_)**	1	1/3
**Incentive policy (C_5_)**	3	1

**Table 6:** Comparison matrix of social criteria.

Social criteria	C_6_	C_7_	C_8_	C_9_
**Traffic condition (C_6_)**	1	5	5	3
**Public facilities condition (C_7_)**	1/5	1	1	1/3
**Impact on nearby residents (C_8_)**	1/5	1	1	1/3
**Impact on traffic congestion (C_9_)**	1/3	3	3	1

**Table 7:** Comparison matrix of ecological criteria.

Ecological criteria	C_10_	C_11_	C_12_
**Natural condition (C_10_)**	1	1/3	1/5
**Pollutant emission (C_11_)**	3	1	1/3
**Sensitivity to pollution (C_12_)**	5	3	1

## Result

The calculations were performed following the procedure mentioned in the section “AHP”. The overall weights of the subcriteria are shown in Table 8. In addition, the results of the consistency test are shown in Table 9.

**Table 8:** Weights of criteria and subcriteria.

Criteria weights	Subcriteria local weights	Total weights (*w*_*j*_)
**Economic criteria (0.5785)**	The price of land C_1_ (0.6370)	0.3685
	Labor criteria C_2_ (0.1047)	0.0606
	Customer distribution C_3_ (0.2583)	0.1494
**Political criteria (0.0567)**	City planning C_4_ (0.2500)	0.0142
	Incentive policy C_5_ (0.7500)	0.0425
**Social criteria (0.2684)**	Traffic condition C_6_ (0.5590)	0.1500
	Public facilities condition C_7_ (0.0955)	0.0256
	Impact on nearby residents C_8_ (0.0955)	0.0256
	Impact on traffic congestion C_9_ (0.2495)	0.0670
**Ecology criteria (0.0964)**	Natural condition C_10_ (0.1047)	0.0101
	Pollutant emission C_11_ (0.2583)	0.0249
	Sensitivity to pollution C_12_ (0.6370)	0.0614

**Table 9:** Consistency test.

	CI	RI	CR	Total CR
**Criteria hierarchy**	0.0390	0.9000	0.0433<0.1	0.0267<0.1
**Economic criteria**	0.0193	0.5800	0.0333<0.1	
**Political criteria**	0.0000	0.0000	0.0000<0.1	
**Social criteria**	0.0145	0.9000	0.0161<0.1	
**Ecology criteria**	0.0193	0.5800	0.0333<0.1	

As seen in Table 9, the consistency tests for both the criteria matrix and the total order meet the requirement. Therefore, the weights of 12 subcriteria are *w* = (0.3685, 0.0606, 0.1494, 0.0142, 0.0425, 0.1500, 0.0256, 0.0256, 0.0670, 0.0101, 0.0249, 0.0614)

### Location evaluation using THOWA

#### Evaluation for alternatives

Group decision making (GDM) is more effective in extracting the real case scenarios of the decision problems to add competitive advantages in a supply chain [21]. In this paper, three experts of a heterogeneous group were invited to participate in the evaluation, which has an advantage over a homogenous group by considering different views [45]. The first expert has a bachelor’s degree in transportation and planning and has been working as a planning department manager in a logistics enterprise for more than 10 years. The second expert has a master’s degree in municipal engineering and has been working as a deputy director general in a municipal planning bureau for more than 15 years. In addition, the third expert has a doctorate degree in logistics systems engineering and has been working as an associate professor in academia for over 15 years. The weights of experts are assigned based on their knowledge and backgrounds [46], that is *v* = (*v*_1_,*v*_2_,*v*_3_) = 0.25,0.35,0.4). The experts evaluated the 4 alternatives against the 12 subcriteria from the perspectives of economy, policy, environment and ecology. The original decision matrixes $R_{k}={\left(r_{ij}^{k}\right)}_{4\times 12},(k=1,2,3)$ as given by those experts are shown in Tables 10–12.

**Table 10:** Original decision matrix R_1_.

	C_1_	C_2_	C_3_	C_4_	C_5_	C_6_	C_7_	C_8_	C_9_	C_10_	C_11_	C_12_
**A_1_**	s_0_	s_6_	s_3_	s_2_	s_4_	s_5_	s_5_	s_0_	s_0_	s_3_	s_4_	s_1_
**A_2_**	s_1_	s_5_	s_2_	s_2_	s_3_	s_4_	s_4_	s_1_	s_1_	s_3_	s_2_	s_0_
**A_3_**	s_2_	s_3_	s_4_	s_4_	s_5_	s_5_	s_4_	s_3_	s_4_	s_5_	s_3_	s_4_
**A_4_**	s_3_	s_1_	s_5_	s_5_	s_5_	s_6_	s_1_	s_6_	s_5_	s_4_	s_1_	s_5_

**Table 11:** Original decision matrix R_2_.

	C_1_	C_2_	C_3_	C_4_	C_5_	C_6_	C_7_	C_8_	C_9_	C_10_	C_11_	C_12_
**A_1_**	s_1_	s_4_	s_6_	s_1_	s_1_	s_5_	s_6_	s_0_	s_5_	s_3_	s_5_	s_1_
**A_2_**	s_3_	s_5_	s_2_	s_2_	s_3_	s_3_	s_4_	s_2_	s_4_	s_2_	s_4_	s_3_
**A_3_**	s_4_	s_4_	s_3_	s_5_	s_4_	s_2_	s_3_	s_4_	s_2_	s_5_	s_3_	s_4_
**A_4_**	s_6_	s_2_	s_1_	s_4_	s_6_	s_4_	s_2_	s_6_	s_1_	s_3_	s_1_	s_6_

**Table 12:** Original decision matrix R_3_.

	C_1_	C_2_	C_3_	C_4_	C_5_	C_6_	C_7_	C_8_	C_9_	C_10_	C_11_	C_12_
**A_1_**	s_0_	s_3_	s_6_	s_5_	s_1_	s_1_	s_6_	s_3_	s_3_	s_3_	s_2_	s_1_
**A_2_**	s_1_	s_2_	s_4_	s_5_	s_4_	s_4_	s_5_	s_4_	s_4_	s_4_	s_3_	s_0_
**A_3_**	s_3_	s_1_	s_3_	s_6_	s_5_	s_5_	s_4_	s_5_	s_5_	s_5_	s_4_	s_2_
**A_4_**	s_5_	s_0_	s_1_	s_4_	s_6_	s_6_	s_3_	s_6_	s_6_	s_6_	s_6_	s_3_

### Decision-making process

The calculations were performed with the method explained in the section “THOWA”. In addition, the original decision matrixes are addressed as follows.

1. Convert any element in the original decision matrix into linguistic 2-tuples, such as translating *S*_0_ into (*S*_0_,0).
2. Aggregate all the converted linguistic 2-tuples of alternative *A*_*i*_ (*i* = 1,2,⋯4) in *R*^*k*^ (*k* = 1,2,3) using the TWA operator (Eq (2)) to obtain the overall evaluation value $y_{i}^{k}$, which represents the overall evaluation of alternative *A*_*i*_ corresponding to expert *E*_*k*_. The results are as follows.
  $$y_{1}^{1}=(S_{2},\;0.0795),\;y_{2}^{1}=(S_{2},0.0013),\;y_{3}^{1}=(S_{3},0.3537),\;y_{4}^{1}=(S_{4},‑0.0169)$$
  $$y_{1}^{2}=(S_{3},0.0188),\;y_{2}^{2}=(S_{3},0.0388),\;y_{3}^{2}=(S_{3},0.3896),\;y_{4}^{2}=(S_{4},0.0888)$$
  $$y_{1}^{3}=(S_{2},‑0.0854),\;y_{2}^{3}=(S_{3},0.0388),\;y_{3}^{3}=(S_{3},‑0.4582),\;y_{4}^{3}=(S_{4},0.2303)$$
3. Aggregate the overall evaluation value $y_{i}^{k}$ (*k* = 1,2,3) using THOWA operator (Eq (3)) to obtain the total evaluation value of alternative *A*_*i*_ (*i* = 1,2,⋯4). Here, the weights of the ordered weighted averaging operators are *ω* = (0.2429 0.5142 0.2429), which are calculated with Eq (4). The weights of the experts are *υ* = (0.25,0.35,0.4). Finally, the total evaluation values y_*i*_ (*i* = 1,2,⋯4) corresponding to alternative A_*i*_ are as follows.
  $$y_{1}=(S_{2},0.2989),\;y_{2}=(S_{3},‑0.1476),\;y_{3}=(S_{3},0.0030)\;,y_{4}=(S_{4},0.1106)$$
4. By comparing the total values of the four alternatives, we find that *y*_4_>*y*_3_>*y*_2_>*y*_1_. Therefore, A_4_ is the optimal location for constructing the new DC.

### Sensitivity analysis

To investigate the influence of criteria weights on the location selection of the DC, a sensitivity analysis was conducted. We conducted four experiments. Table 13 presents the details of the experiments.

**Table 13:** Experiments for sensitivity analysis.

Expt No.	Weights of criteria				Weights of subcriteria											
	economic	political	social	ecological	C1	C2	C3	C4	C5	C6	C7	C8	C9	C10	C11	C12
**1**	0.7	0.1	0.1	0.1	0.4459	0.0733	0.1808	0.0250	0.0750	0.0559	0.0095	0.0095	0.0250	0.0105	0.0258	0.0637
**2**	0.1	0.7	0.1	0.1	0.0637	0.0105	0.0258	0.1750	0.5250	0.0559	0.0095	0.0095	0.0250	0.0105	0.0258	0.0637
**3**	0.1	0.1	0.7	0.1	0.0637	0.0105	0.0258	0.0250	0.0750	0.3913	0.0669	0.0669	0.1747	0.0105	0.0258	0.0637
**4**	0.1	0.1	0.1	0.7	0.0637	0.0105	0.0258	0.0250	0.0750	0.0559	0.0095	0.0095	0.0250	0.0733	0.1808	0.4459

It can be seen from Table 13 that in the four experiments, we set each criterion as the highest one-by-one and left the low and equal weights for the remaining criteria. To highlight the importance of the criteria investigated, we designed the highest weight of a criterion as 0.7, and the remaining criteria as 0.1. Furthermore, the corresponding weights of the subcriteria were recalculated simultaneously.

**Fig 3:**
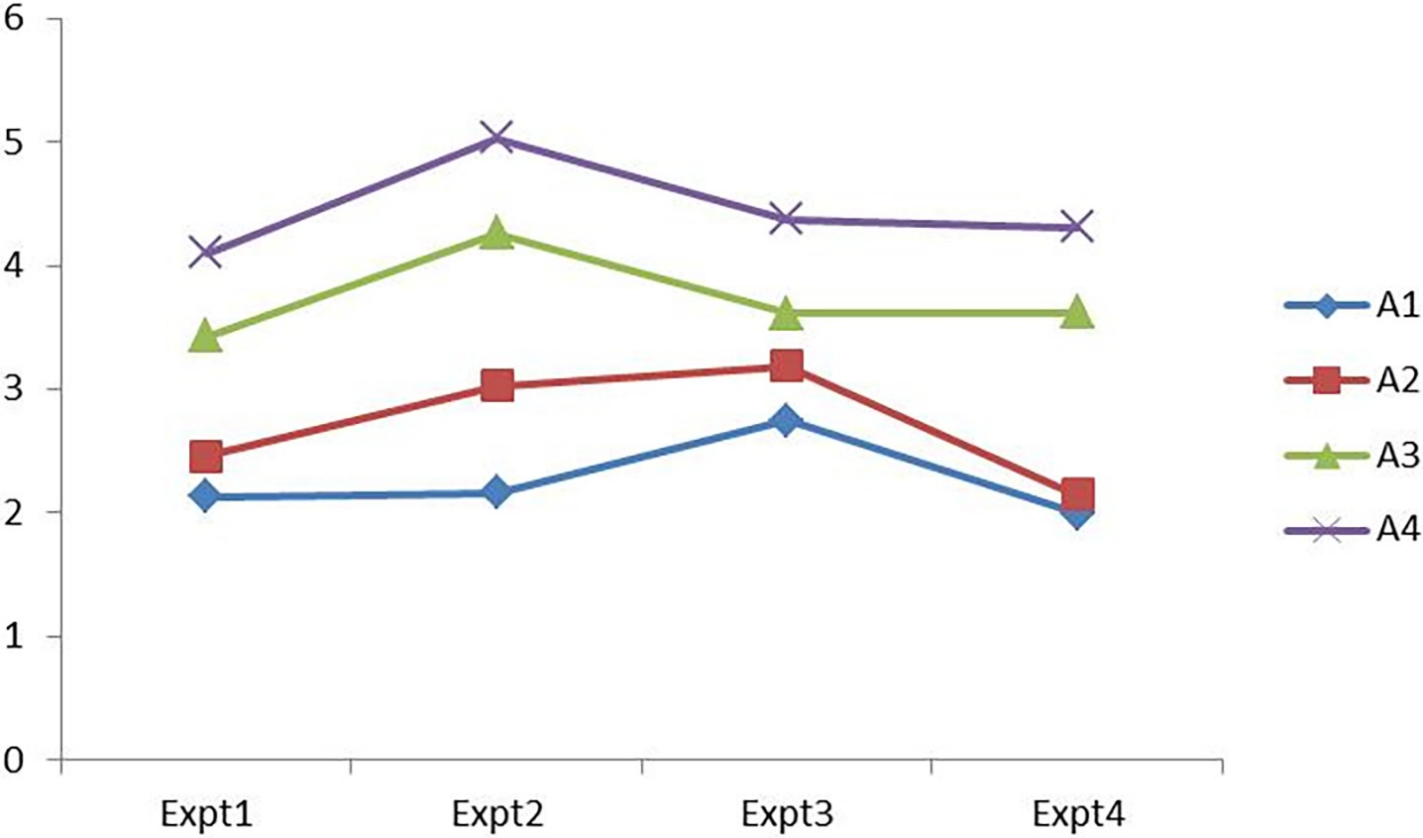
Results of sensitivity analysis.

The results are presented in Fig 3, in which the value of the linguistic 2-tuple was converted into a numerical value. In the four experiments, location A_4_ has the highest score, and the ranking order of the four alternatives has always been A_4_>A_3_>A_2_>A_1_, which illustrates that A_4_ emerges as the optimal alternative considering the 4 criteria and 12 subcriteria. Therefore, it indicates that the location decision is insensitive to the benefit criteria weights, and our method has high robustness.

## Conclusion

In this paper, a generic framework for the location selection of a DC under a fuzzy environment has been proposed. The proposed method involves four steps. In step 1, we identified the criteria and subcriteria for evaluating alternatives in megacities based on the literature review, analysis of megacities’ characteristics, and opinions of experts. The results are four categories of criteria, namely, economic, political, social and ecological. In step 2, AHP was adopted to calculate the weights of the subcriteria combining the evaluation information from logistics experts. In step 3, the experts of a heterogeneous group provided ratings for the alternatives. THOWA was used to aggregate the ratings and produced an overall score for measuring the performance of the alternatives. Notably, we improved the THOWA operator by applying a more advanced technique for computing the weights of ordered weighted average operators to reduce the influence of biased evaluation information on the results more effectively. In step 4, the sensitivity analysis was performed to identify the effect of the criteria weights on the decision-making process. The results of the sensitivity analysis demonstrated that our method has high robustness. A case was conducted to demonstrate the validity and practicability of the proposed methodology. The strength of our work is that we provide an effective and practical approach (AHP+THOWA) for location selection for DCs in Chinese megacities for municipal planning departments under limited quantitative information.

The limitation of this paper is that the weaknesses of AHP, such as its axiomatic foundation, the potential problems of rank reversal and arbitrary ranking, have not been considered adequately. Hence, we will explore a more accurate and objective method to calculate the weights of criteria in future research.

Finally, another intended direction of our future work is to perfect the evaluation criteria system by adding additional factors, such as reputation and security, making it more suitable for the location selection of DCs in megacities.

## Supporting information

S1 File Supporting information for sensitivity analysis. (DOCX)
