# Supplementary material for: A hybrid approach based on the analytic hierarchy process and 2-tuple hybrid ordered weighted averaging for location selection of distribution centers
Source: PLoS One. 2018 Nov 8;13(11):e0206966. doi: 10.1371/journal.pone.0206966 (PMC6224092; doi:10.1371/journal.pone.0206966)
Supplement: S1 File — (DOCX) [file pone.0206966.s001.docx]

**Supporting information**

All the information of section “Numerical illustration” has been listed in the revised manuscript. But the information of section “Sensitive analysis” has been omitted partly in the revised manuscript. Hence, we provide the information in this file. As shown as following tables.

S1A Table. The overall evaluation value$y_{i}^{k}$ in experiment 1

| **Evaluation value** | **E1** | **E2** | **E3** |
| --- | --- | --- | --- |
| **A1** | ( S_2_, -0.1424) | ( S_3_, -0.3904) | ( S_2_, -0.1321) |
| **A2** | ( S_2_, -0.1718) | ( S_3_,- 0.0192) | ( S_2_, 0.2692) |
| **A3** | ( S_3_, 0.1406) | ( S_4_, -0.3428) | ( S_3_, 0.2515) |
| **A4** | ( S_4_, -0.2718) | ( S_4_, 0.3169) | ( S_4_, -0.0599) |

S2B Table. The overall evaluation value$y_{i}^{k}$ in experiment 2

| **Evaluation value** | **E1** | **E2** | **E3** |
| --- | --- | --- | --- |
| **A1** | ( S_3_, 0.1158) | ( S_2_, -0.3538) | ( S_2_, -0.0505) |
| **A2** | ( S_2_, 0.4720) | ( S_3_, -0.1388) | ( S_4_, -0.3086) |
| **A3** | ( S_4_, 0.4178) | ( S_4_, -0.0378) | ( S_5_, -0.2729) |
| **A4** | ( S_5_, -0.2562) | ( S_5_, 0.0431) | ( S_5_, 0.1741) |

S3C Table. The overall evaluation value$y_{i}^{k}$ in experiment 3

| **Evaluation value** | **E1** | **E2** | **E3** |
| --- | --- | --- | --- |
| **A1** | ( S_3_, -0.0211) | ( S_4_, -0.1839) | ( S_2_, 0.0506) |
| **A2** | ( S_3_,-0.3997) | ( S_3_, 0.1596) | ( S_3_, -0.4016) |
| **A3** | ( S_4_, 0.2454) | ( S_3_, -0.2158) | ( S_5_, -0.4808) |
| **A4** | ( S_5_, -0.0935) | ( S_4_, -0.3064) | ( S_5_, 0.3013) |

S4D Table. The overall evaluation value$y_{i}^{k}$ in experiment 4

| **Evaluation value** | **E1** | **E2** | **E3** |
| --- | --- | --- | --- |
| **A1** | ( S_2_, 0.2064) | ( S_2_, 0.3918) | ( S_2_, -0.3699) |
| **A2** | ( S_1_, 0.3204) | ( S_3_, 0.1024) | ( S_2_, -0.1424) |
| **A3** | ( S_4_, -0.1244) | ( S_4_, -0.2800) | ( S_3_, 0.2755) |
| **A4** | ( S_4_, 0.0610) | ( S_4_, 0.3797) | ( S_4_, 0.3275) |

S5E Table. The total evaluation value of alternative$A_{i}$ in the 4 experiments

| **Total evaluation value** | **Experiment 1** | **Experiment 2** | **Experiment 3** | **Experiment 4** |
| --- | --- | --- | --- | --- |
| **A1** | ( S_2_, 0.1278) | ( S_2_, 0.1604) | ( S_3_, -0.2560) | ( S_2_, -0.0092) |
| **A2** | ( S_2_, 0.4602) | ( S_3_, 0.0294) | ( S_3_, 0.1853) | ( S_2_, 0.1488) |
| **A3** | ( S_3_, 0.4283) | ( S_4_, 0.2639) | ( S_4_, -0.3843) | ( S_4_, -0.3799) |
| **A4** | ( S_4_, 0.1026) | ( S_5_, 0.0269) | ( S_4_, 0.3740) | ( S_4_, 0.3074) |
